# A case-crossover study of alcohol consumption, meals and the risk of road traffic crashes

**DOI:** 10.1186/1471-2458-9-316

**Published:** 2009-09-01

**Authors:** Stefano Di Bartolomeo, Francesca Valent, Rodolfo Sbrojavacca, Riccardo Marchetti, Fabio Barbone

**Affiliations:** 1 Agenzia Regionale della Sanità del Friuli Venezia Giulia/Cattedra di Epidemiologia, DPMSC, Università degli Studi di Udine, Via Pozzuolo 330, 33100 Udine, Italy; 2 SOC Istituto di Igiene ed Epidemiologia, Azienda Ospedaliero-Universitaria di Udine, Via Colugna 50, 33100 Udine, Italy; 3 S.O.C. Pronto Soccorso, Azienda Ospedaliero-Universitaria di Udine, Pzza SM Misericordia, 33100 Udine, Italy; 4 Cattedra di Epidemiologia, DPMSC, Università degli Studi di Udine, Via Colugna 50, 33100 Udine, Italy; 5 Cattedra di Epidemiologia, DPMSC, Università degli Studi di Udine and SOC Istituto di Igiene ed Epidemiologia, Azienda Ospedaliero-Universitaria di Udine, Via Colugna 50, 33100 Udine, Italy

## Abstract

**Background:**

The case-crossover (CC) design has proved effective to investigate the association between alcohol use and injuries in general, but has never been applied to study alcohol use and road traffic crashes (RTCs) specifically. This study aims at investigating the association between alcohol and meal consumption and the risk of RTCs using intrapersonal comparisons of subjects while driving.

**Methods:**

Drivers admitted to an Italian emergency room (ER) after RTCs in 2007 were interviewed about personal, vehicle, and crash characteristics as well as hourly patterns of driving, and alcohol and food intake in the 24 hours before the crash. The odds ratio (OR) of a RTC was estimated through a CC, matched pair interval approach. Alcohol and meal consumption 6 and 2 hours before the RTC (case exposure window) were compared with exposures in earlier control windows of analogous length.

**Results:**

Of 574 patients enrolled, 326 (56.8%) reported previous driving from 6 to 18 hours before the RTC and were eligible for analysis. The ORs (mutually adjusted) were 2.25 (95%CI 1.11-4.57) for alcohol and 0.94 (0.47-1.88) for meals. OR for alcohol was already increased at low (1-2 units) doses - 2.17 (1.03-4.57) and the trend of increase for each unit was significant - 1.64 (95%CI 1.05-2.57). In drivers at fault the OR for alcohol was 21.22 (2.31-194.79). The OR estimate for meal consumption seemed to increase in case of previous sleep deprivation, 2.06 (0.25-17.00).

**Conclusion:**

Each single unit of acute alcohol consumption increases the risk of RTCs, in contrast with the 'legal' threshold allowed in some countries. Meal consumption is not associated with RTCs, but its combined effects with sleepiness need further elucidation.

## Background

Driver-related behavioural factors are major contributors to the occurrence of road traffic crashes (RTCs), [1,2] that in turn are the commonest cause of injury fatalities worldwide [3]. Among these factors, alcohol consumption plays an important role. In fact, 30-40% of driver deaths in the European Union result from driving under the influence of alcohol [4].

The association between alcohol and injuries has been explored in depth in the recent years, shedding light on aspects like the relative contribution of acute vs. chronic alcohol use and of various levels of alcohol consumption. One study has shown that alcohol use in the 6 hours prior to injury is associated with an increased risk of injury and postulated a dose-response effect [5]. Other studies have demonstrated that the effect of alcohol is stronger for acute exposure than for long-term exposure and that the risk is significant even at a low (1-2 units) consumption level [6-8].

All these studies benefited from the case-crossover design, [9,10] that has proved to be effective in estimating the risk of sudden events associated with transient exposures with short effect, such as acute alcohol consumption. However, all these studies investigated injuries of any cause - and therefore did not take into account the fact that, when RTCs are considered, the target person times at risk are driving times. In fact, it would be impossible for the drivers to be involved in collisions while not driving [10] and thus intrapersonal comparative analyses should consider only periods while the subjects are driving.

Finally, alcohol is often consumed during meals, which may both delay its absorption [11] and cause sleepiness [12] which is an additional trigger of RTCs [13]. To investigate the acute effects of alcohol and meal consumption on the risk of RTCs, we conducted a case-crossover study that compared driving periods among RTC cases recruited for one year at the largest emergency room (ER) of Friuli Venezia Giulia Region, Italy.

## Methods

### Study subjects

Subjects for this case-crossover study were recruited at the ER of the Hospital of Udine, North-Eastern Italy, from March 12^th^, 2007, to March 11^th^, 2008. They were injured drivers (including motorcyclists and cyclists) who presented for care to the ER after being involved in a RTC. Drivers were eligible if they were ≥ 14 years of age, alive at the time of arrival at the ER, and sufficiently proficient in Italian to be interviewed. Subjects were included in the study if they (or their parents in case of drivers <18 years of age) provided written consent to participate in the research and if an interview was possible within 36 hours from the time of RTC.

Potential study subjects were identified by ER personnel (triage nurses) who alerted trained interviewers who systematically covered selected 12-hour (weekends and nights) or 6-hour (days Monday to Friday) shifts at the ER (covered hours = 3432). The interviewers approached the eligible drivers and proposed the participation in the research, without hindering or delaying diagnostic and care activities. When possible, subjects consenting to the study were interviewed directly at the ER. The study was approved by the ethics committee of the "Azienda Ospedaliero-Universitaria" of Udine, Italy.

### Data collection

Data were collected through a semi-structured questionnaire administered by a limited number of interviewers who had received specific training. Within a few days from the interview, the filled-in forms were checked for completeness and quality and interviewers or subjects were re-contacted if clarifications were needed.

The questionnaire collected information on socio-demographic characteristics of the driver and driving habits, characteristics of the vehicle and the RTC, usual alcohol consumption and drink & drive habits, and other potential risk factors not reported in this article. Alcohol, food intake and driving were assessed in each of the 24 hours before the RTC. Additionally, sleep was tracked in the 48 hours before the RTC. Each interview lasted approximately 30 minutes.

### Statistical analysis

In the case-crossover design each case acts as his/her own control and therefore interpersonal confounding for factors that do not vary suddenly with time is eliminated. The length of the hazard period was chosen based on Maclure and Mittleman's suggestion (i.e. the one that maximizes the relative risk) [10]. It was set at 6 hours for alcohol and 2 hours for meal intake. Therefore, the windows for alcohol and meals were 6 and 2 hours respectively. The positioning of the control exposure-window was based on the fact that in collision studies the target person times at risk are driving times. In fact, it would be physically impossible for the drivers to be involved in collisions when not driving. Therefore, only subjects who reported driving at the control window were included, as emphasized by Maclure and Mittleman [10] and done by McEvoy et al. [14] and Redelmeier and Tibshirani [15]. In order to maximise the number of these subjects, the control window was not at a fixed distance in time from the case window across all subjects (e.g. the same time of the previous day or the same time and day of the previous week) as customary in previous literature [5-8]. The control window was the period that preceded the first episode of driving that in turn preceded the case window. In other words, the control period was any 6 hours for alcohol and any 2 hours for meals before the first driving reported between 6 and 18 hours prior to the RTC. If a subject had more than one eligible driving period, the first one was analyzed. A driving episode was eligible only if it concerned the same type of vehicle as the one of the RTC, according to the classification shown in table 1.

**Table 1:** Characteristics of all the participants to the study (N = 574) and of the subgroup (N = 326) admitted to the case-crossover analysis^a^.

	All subjects	Subjects in case-crossover analysis
Gender, male, No. (%)	301 (52.4)	175 (53.7)
Age, mean ± standard deviation (median)	39.8 ± 15.2 (38)	38.2 ± 13.2 (38)
Level of education, No. (%)		
Elementary school	41 (7.1)	12 (3.7)
Middle school	175 (30.5)	98 (30.1)
High school	271 (47.2)	165 (50.6)
University	86 (15.0)	51 (15.6)
Occupation, No. (%)		
Employed	412 (71.8)	263 (80.7)
Student	49 (8.5)	23 (7.1)
Housewife	24 (4.2)	9 (2.8)
Retired	72 (12.5)	24 (7.4)
Unemployed	17 (3.0)	7 (2.1)
Marital status, No. (%)		
Married (spouse/husband present)	250 (43.5)	142 (43.7)
Widowed, divorced or separated	76 (13.2)	36 (11.0)
Single (never married)	245 (42.7)	147 (45.2)
Nationality, No. (%)		
Italian	522 (90.4)	296 (91.4)
Foreign, European Union	9 (1.6)	5 (1.5)
Foreign, Non European Union	37 (6.4)	23 (7.1)
Driving license held for, No. (%)		
No license	31 (6.3)	13 (4.1)
<1 year	11 (2.2)	6 (1.9)
1-4 years	57 (11.6)	38 (12.1)
≥ 5 years	392 (79.8)	255 (81.7)
Type of vehicle driven during the crash, No. (%)		
Car	367 (64.4)	226 (69.3)
Motorcycle	111 (19.5)	64 (19.6)
Bicycle	82 (8.5)	28 (8.6)
Van	7(1.2)	5 (1.5)
Truck	3 (0.5)	3 (0.9)
Vehicle of the crash is of the type most frequently driven, No. (%)	477 (83.1)	254 (84.4)
Weekend^b^, No. (%)	191 (33.3)	103 (31.6)
Night^b, c^, No. (%)	60 (10.4)	44 (13.5)
Unfavourable weather (rain, snow, fog, hail or strong wind)^b^, No. (%)	105 (18.3)	64 (19.6)
Uneven, wet, or dirty road surface^b^, No. (%)	196 (34.1)	111 (34.0)
Type of crash, No. (%)		
Two motor vehicle, head-on	26 (4.5)	14 (4.3)
Two motor vehicle, side	171 (29.8)	100 (30.8)
Two motor vehicle, rear end	213 (37.1)	126 (38.8)
One vehicle (rollover or impact against fixed obstacle)	78 (13.6)	49 (15.0)
Fall from vehicle	77 (13.4)	32 (9.8)
Other	6 (1.0)	4 (1.2)
Previous experience of the route of crash, No. (%)		
Habitual or well known	380 (66.2)	222 (68.1)
Already done but not well known	170 (29.6)	93 (28.5)
Entirely new	22 (3.8)	11 (3.4)
Driver at fault (self-rated), No. (%)		
Yes	151 (26.3)	80 (24.7)
Partially	55 (9.6)	30 (9.3)
No	364 (63.9)	214 (66.0)

^a ^Percentages may not add too 100% due to missing values. ^b ^Referred to the case window. ^c ^Night is approximated as from 6.00 pm to 6.59 am from October to March and from 8.00 pm to 5.59 am from April to September

The analyses were conducted following the matched pair interval approach. Conditional logistic regression was used to estimate the Odds Ratio (OR) of RTC. As in previous studies, [5-8] the main exposure variable was consumption of at least 1 unit of alcohol (i.e., 10 milliliters of ethanol, approximately equivalent to 1 small glass of wine, one can of lager beer or a bar measure - 30 ml - of spirit) in the hazard period vs. none. A dose-response analysis of alcohol consumption was also conducted (0, 1-2, and 3 or more units). The exposure to a meal was defined as the intake of a regular or heavy meal while no food consumption or the consumption of only a light meal or snacks were used as the reference category.

Our choice of the control window may imply some intrapersonal confounding by clock time because the baseline risk of having a RTC while driving is likely to have a circadian variation (e.g. higher by night time because of dark or at rush hours because of traffic). At the same time, our case window and control window were separated by an interval that was unfixed, as already explained, and was invariably a fraction of this circadian rhythm (e.g. one window could be in daylight and the other in night time). Therefore, an adjustment was done for time of day in 6 categories: 5:00-8:59, 9:00-12:59, 13:00-16:59, 17:00-20:59, 21:00-00:59, 01:00-04:59. To check if these categories were adequate or implied some residual confounding a model was also run using a series of 23 indicator variables coding for the 24 hours of the day, as suggested by Mittleman et al. [16]. Stratified analyses were conducted to explore the modification of risks by possible factors.

The interaction between alcohol and meals was studied by introducing a product term of the 2 exposures in the model. In a further attempt to isolate the pure effects of meal intake from the possible modification by the effects of alcohol, an analysis comprised of only subjects that had not consumed alcohol neither in the case nor in the control windows was performed.

Further analyses were conducted to verify a possible interaction of sleep deprivation with either alcohol or meals. Sleep deprivation was defined as a binary variable corresponding to having slept in the 24 hours before the case or control windows a number of hours lower than declared as usual by the patient. Two models were then run containing time bands and a set of dummies expressing all the possible combinations of sleep deprivation and either alcohol or meal consumption.

## Results

During the 12 months of the study, 877 injured drivers arrived at the ER during our recruitment shifts. Of them 574 (65.5%) were enrolled; 100 subjects were injured too seriously to be interviewed, 95 refused to sign the informed consent, 40 were lost because of the contemporary arrival or more than one driver, 24 had no time to be interviewed, 22 did not understand Italian, 5 were aged < 18 years with no parental consent, 3 were both too intoxicated (presumably by alcohol) to be interviewed in ER and not available for a later interview, and 14 did not meet some other inclusion criteria. Most interviews took place in the ER (N = 546, 95.1%) soon after the subject's arrival. Table 1 illustrates the main characteristics of participant subjects and of the RTCs in which they had been involved; the third column displays the same information restricted to the subjects involved in the crossover analysis, i.e., those who had driven at least once between 6 and 18 hours before the crash.

Of all the 574 cases, 326 (56.8%) reported driving at some stage between 6 and 18 hours before the RTC and underwent the pair-matched analysis. The distribution of pairs according to both alcohol and meal-intake exposure is shown in table 2. The unadjusted OR for alcohol consumption was 1.33 (95%CI: 0.69-2.60) and for meal intake was 1.08 (95%CI: 0.59-2.00).

**Table 2:** Unadjusted matched pair analysis of alcohol and food intake exposure prior to road traffic crash (RTC) and prior to the previous occurrence of driving.

Alcohol consumption in the 6 hours prior to crash	Alcohol consumption in the 6 hours prior to previous episode of driving		
	yes	no	total
yes	3	24	27
no	18	281	299
total	21	305	326
OR = 1.33 (95%CI: 0.69-2.60)			

Meal intake in the 2 hours prior to crash	Meal intake in the 2 hours prior to previous episode of driving		
	yes	no	total
yes	4	25	29
no	23	276	297
total	27	301	326
OR = 1.08 (95%CI: 0.59-2.00)			

Distribution of discordant window pairs, odds ratio (OR) of RTC and 95% confidence intervals (95%CI).

The results of the full model including the two exposures and the time of day are shown in table 3, the ORs were 2.25 (95%CI: 1.11-4.57) for alcohol and 0.94 (95%CI: 0.47-1.88) for meals.

**Table 3:** Odds Ratios (OR) and 95% confidence intervals (CI) for alcohol and food intake with mutual and time-of-day adjustment.

Variable	OR	95%CI
Alcohol consumption in the previous 6 hours	2.25	1.11-4.57
Meal intake in the previous 2 hours	0.94	0.47-1.88
Time band 05:00-08:59	1	
Time band 09:00-12:59^a^	1.63	0.99-2.68
Time band 13:00-16:59^a^	2.08	1.30-3.33
Time band 17:00-20:59^a^	0.79	0.52-1.19
Time band 21:00-00:59^a^	0.30	0.14-0.66
Time band 01:00-04:59^a^	0.91	0.30-2.77

^a ^reference category is time band 05:00-08:59

When the adjustment for time of day was done on an hourly basis the results were similar (alcohol OR 2.51, 95%CI: 1.17-5.37; meals OR 0.84, 95%CI: 0.38-1.89).

Table 4 shows the OR for alcohol and meals from the full model stratified by potential effect modifiers. Alcohol use in the previous 6 hours and being at fault strongly interacted in triggering the crash. A lower level of education also increased the risk - OR for higher education 2.16 (95% CI 0.91-5.14) and OR for lower education 5.99 (95% CI 1.04-34.57). Some other subgroups of subjects exposed to alcohol (drivers who held a license for less than 5 years, females, the younger than 25 years and those with lower habitual consumption) show a possible, non significant, association with a greater risk of RTC.

**Table 4:** Odds ratios (OR) and 95% confidence intervals (CI) for alcohol and meal intake stratified by potential effect modifiers.

Possible modifier	Exposure			
	
	Alcohol consumption		Meal intake	
	
	OR^a^	95%CI	OR^a^	95%CI
Gender				
Male (N = 175)	1.83	0.77-4.35	1.72	0.63-4.66
Female (N = 151)	4.58	0.81-25.93	0.63	0.20-1.96
Age				
<25 years (N = 57)	5.00	0.43-57.31	0.55	0.08-3.59
≥ 25 years (N = 269)	2.10	0.99-4.44	0.98	0.45-2.11
Level of education (high school or higher)				
No (N = 110)	5.99	1.04-34.57	2.09	0.41-10.60
Yes (N = 216)	2.16	0.91-5.14	0.64	0.28-1.47
Vehicle				
Car, van, truck (N = 234)	2.55	1.04-6.25	0.81	0.36-1.82
Motorcycle (N = 64)	2.68	0.50-14.26	1.02	0.15-6.82
Bicycle (N = 28)	2.99	0.09-99.06	0.83	0.02-31.40
Driving license held for (only motor-vehicle drivers)				
<5 years (N = 41)	9.99	0.46-217.78	0.61	0.05-7.34
≥ 5 years (N = 257)	1.96	0.89-4.29	0.79	0.36-1.73
Driver at fault				
Yes (N = 80)	21.22	2.31-194.79	0.82	0.16-4.18
Yes or partially (N = 110)	10.25	2.42-43.33	0.95	0.24-3.77
No (N = 214)	1.12	0.47-2.69	0.82	0.36-1.85
Habitual consumption of 12 or more drinks a month				
No (N = 211)	4.17	0.72-24.16	0.77	0.29-2.05
Yes (N = 115)	1.63	0.67-3.96	1.31	0.47-3.64

^a ^From conditional logistic regression models including alcohol consumption, meal intake and time-of-day.

For alcohol, the dose-response analysis (full model) showed evidence of effect (OR 2.17, 95%CI: 1.03-4.57) even at low level of exposure (1-2 drink units), while the OR for an intake greater than 2 units was 3.02 (95%CI: 0.28-31.74). The OR for the interaction term alcohol consumption*meal consumption was 1.12 (95%CI: 0.18-6.72). When considering only subjects who had not consumed alcohol the OR of meal intake was 0.78 (95%CI: 0.34-1.77). Compared to sleep deprivation alone, concurrent alcohol consumption and sleep deprivation was associated with a OR of 2.98 (95%CI: 0.43-20.62), whereas concurrent meal consumption and sleep deprivation was associated with a OR of 2.06 (95%CI: 0.25-17.00).

## Discussion

### Main findings

This study shows that consumption of any quantity of alcohol within 6 hours prior to driving is associated with a 2.25-fold increase in RTC risk. In the previous studies that adopted the same hazard period and presented analyses stratified for mechanism of injury, [5,6,8] alcohol was associated with a OR of RTC of 2.7, 5.0 and 3.9, respectively. However, because these studies included all types of injuries, they did not adopt the correct definition of time at risk, i.e., did not restrict the time at risk to driving time.

The effect of alcohol is present even at intake of 1-2 units - OR = 2.17, 95%CI: 1.03-4.57. This would most likely correspond to a blood level considered legal according to the Italian legislation (< 50 g/l). This is a flaw of several current laws worldwide that had already been pointed out [17].

Although based on small numbers, certain subgroups of drivers showed stronger effects of alcohol on RTCs (table 4). The interpretation of these findings is difficult, also because some of these variables could be proxy of each other. A possible explanation could be that the effects are larger when drinking occurs in the unaccustomed and/or in a sporadic, binge-like fashion. This would be consistent with a previous report [5] but not with another one [8]. Moreover, the results about usual consumption should be taken with caution because it has been shown [18] that the simple estimation of usual consumption may mask a wide range of drinking patterns (i.e., low and steady or infrequent and heavy) that carry different risks. Finally, because of both the definition of alcohol exposure in our study (i.e. any quantity in the 6 hours) and the likely existence of a dose-effect, the modification of risk could be spurious, i.e. merely reflect a higher intake on average in the exposure windows. The sharp increase in effect with increasing culpability adds to the reliability of our methods.

As for meal intake, despite the reasonable and common belief that its detrimental effects on cognitive functions [12,19,20] could increase the risk of RTCs, this association is not confirmed on the whole by our study, the first one we are aware of. Nor did the consumption of meals seem to influence the risk of alcohol assumption, perhaps because this interaction can go in 2 opposite directions, as already explained. The results of the analysis of the interaction with sleep deprivation show a doubling of the point estimate of risk (2.06), but are limited in precision (95%CI: 0.25-17.00). Therefore it seems wise not to question the common belief that driving should be avoided after heavy meals until more evidence is gathered.

### Methodology and limitations

The case-crossover design offers the conspicuous advantage of eliminating interpersonal confounding and problems in the selection of control groups. Its previous applications to the study of risk factors for RTCs have been successful [13,14,21]. However, this particular application requires the fulfilment of the 'driving opportunity' criterion, i.e. a special definition of time at risk that should be 'time while driving'. Since previous studies targeted all injuries, they did not restrict the hazard period to time while driving. Moreover, in these studies not only drivers but all injured patients were included. It is questionable though if alcohol assumption by a transported passenger can have any role in the causation of a RTC as indirectly shown also by our data on effect modification by culpability.

The fulfilment of the 'driving opportunity' criterion is, however, not without shortcomings. First, it greatly reduces the number of interviewed patients who remain eligible. For example, the percentage of subjects who reported driving was 16% at minus 6 hours, 34% at minus 1 hour and 40% at minus 24 hours. Therefore, we chose a 'flexible' control window, i.e. the first prior episode of driving between 6 and 18 hours from the crash. In this way we were able to include the highest percentage (56.8) of available subjects, while allowing for a full 6 hours of exposure window for control periods, that are all positioned within 24 hours from the crash.

Secondly, it has to be recognized, that the 'driving opportunity' criterion is truly fulfilled when the subject drives for the same fraction of time within the case and the control window. Similarly, other conditions that may have affected the risk of RTC while driving (e.g. weather and road conditions, traffic congestion etc.) had to be arbitrarily assumed as similar in the 2 windows. The alternative would have been to record this information in both windows and account for them in the analysis but this would have been very impractical and difficult.

One could wonder if the requirement of driving implies the possibility of selection bias. This bias occurs when the selection criteria are associated with the exposure, a fact that may occur in the case of drinking and driving. However, the case-crossover design should prevent this because the same subject - with his/her unique profile of habitual drinking and driving and eating and driving - provides both the case and control information. Nevertheless, selection and report bias may affect case-crossover studies on alcohol. Patients who have a RTC under the influence of alcohol could be more likely to avoid ER admission in case of trivial injuries, deny consent to the interview and underreport the assumption of alcohol before the crash, for fear of legal consequences. Moreover, in case of severe alcohol intoxication the interview may be impossible, an event that occurred 3 times in our series. All these possibilities, however, would bias toward the null the estimation of alcohol effects. Therefore, the effects of alcohol are likely to be larger than we found. On the other hand, disloyal scaling-down of alcohol consumption could partly explain the increased risk we found at low doses.

Another limitation of our estimates is that they cannot be generalized to severely injured patients because they could not be interviewed and therefore are not represented. Once again, however, this is likely to have led to an underestimation of the global risk of alcohol consumption because there is evidence of increased effect with increasing injury severity [6].

Finally, recall bias is also an issue when past exposure measurement is based on interviews. It is known that the accuracy of recall in humans significantly depends on the time interval between the event and the time of its assessment: the longer the interval, the higher the probability of incorrect recalls [22]. It is also known, from case-control studies that data, even about irrelevant exposures, are often remembered better by cases or/and underreported by controls [23]. If this applied also to CC studies and led to an underreporting of exposures in control windows, the risk estimates would be inflated. Conversely, if recall bias caused only a loss of precision of the timing - not quantity - of exposures, the effects would probably be minor because they would still be included in the hazard periods of the investigated risk factors that are relatively long, especially for alcohol.

## Conclusion

This study confirms that recent alcohol consumption, even at low doses, is a risk factor for RTCs that doubles in case of consumption of any quantity in the 6 hours prior to driving.

The methodology adopted should have avoided limitations of previous case-crossover studies on alcohol that targeted injuries as a whole, although at the price of decreased efficiency.

Possible limitations of the study, if present, should have underestimated the real risks of alcohol. On the other hand, driving after the intake of a regular or heavy meal does not increase the risk of RTCs on the whole, however one cannot dismiss the idea that it does so in case of pre-existing sleepiness.

## Competing interests

The authors declare that they have no competing interests.

## Authors' contributions

SDB participated in the design, coordination and quality assurance of the study, performed the statistical analyses, drafted the manuscript and approved the final version. FV conceived the study, participated in its design, coordination and quality assurance, participated in the statistical analyses, helped to draft the manuscript and approved the final version. RM inputted the data, participated in the coordination and quality assurance of the study, contributed to draft the manuscript and approved the final version. RS participated in the acquisition of data, contributed to revise the article and approved the final version. FB conceived the study, participated in the design of the study, overviewed the statistical analyses, revised the manuscript critically for important intellectual content and approved its final version.

## Pre-publication history

The pre-publication history for this paper can be accessed here:


